# Plant Spliceosomal Introns: Not Only Cut and Paste

**DOI:** 10.2174/138920208784533629

**Published:** 2008-06

**Authors:** L Morello, D Breviario

**Affiliations:** Istituto Biologia e Biotecnologia Agraria, Via Bassini 15, 20133 Milano, Italy

## Abstract

Spliceosomal introns in higher eukaryotes are present in a high percentage of protein coding genes and represent a high proportion of transcribed nuclear DNA. In the last fifteen years, a growing mass of data concerning functional roles carried out by such intervening sequences elevated them from a selfish burden carried over by the nucleus to important active regulatory elements. Introns mediate complex gene regulation *via *alternative splicing; they may act in *cis* as expression enhancers through IME (intron-mediated enhancement of gene expression) and in *trans* as negative regulators through the generation of intronic microRNA. Furthermore, some introns also contain promoter sequences for alternative transcripts. Nevertheless, such regulatory roles do not require long conserved sequences, so that introns are relatively free to evolve faster than exons: this feature makes them important tools for evolutionary studies and provides the basis for the development of DNA molecular markers for polymorphisms detection. A survey of introns functions in the plant kingdom is presented.

## INTRODUCTION

Thirty years have elapsed since the discovery of interrupted genes in Adenovirus2 mRNA, then defined as “amazing rearrangements” [1]. Now, the exon-intron organization of eukaryotic genes and the splicing machinery are no more a surprise but are still fascinating in the light of the newly uncovered roles played by introns in controlling gene expression.

Introns represent a large proportion of the vast genomic noncoding DNA from which only a small percentage (1-2%) of coding DNA sequences differentiate. Introns length and density across the genomes of different eukaryotes vary enormously, from very few introns in some fungi such as *S. cerevisiae* to hundreds of thousands in higher plants and animals. Nevertheless, their number is not proportional to organism complexity: i.e. *Drosophila melanogaster *and *Caenhorabditis elegans* have about the same percentage of intronic DNA (29.1% and 30.4%) and number of introns per gene (4.22 and 5.46 respectively) [2**]. Intron density has also been tentatively correlated with population size [3] or to recombination frequencies [4].

Introns can be no more regarded as junk DNA, in the light of the increasing amount of data provided by genome sequencing projects and because of the discovery of regulatory functions that can be attributed to them. If their presence has certainly contributed, early in eukaryotic evolution, to increase the number of protein structures through genome rearrangements and exon shuffling [5], introns still offer enormous plasticity to gene expression, through alternative splicing that greatly increases the cell transcriptional and translational output, a phenomenon whose dimension has been highlighted in recent years.

Introns neutrality in evolution, apparently implicit in the overall high variability of their sequences, has to be clearly reconsidered. In fact, evidence for the existence of some selective constraints in noncoding DNA regions including introns, has emerged by cross-comparison of genomic sequences of closely related *Drosophila* species [6] or analyses of duplicated regions within the rice genome [7]. Selective constraints are identified by the fact that their evolutionary divergence is reduced relative to a neutrally evolving sequence and this may be due to functional constraints. Moreover, short regions of conserved noncoding sequences (CNS) have been recently found among cultivated cereal genomes [8] and in duplicated regions of the Arabidopsis genome [9]. It has been shown that some of these CNSs contain functional elements.

**Fig. (1):**
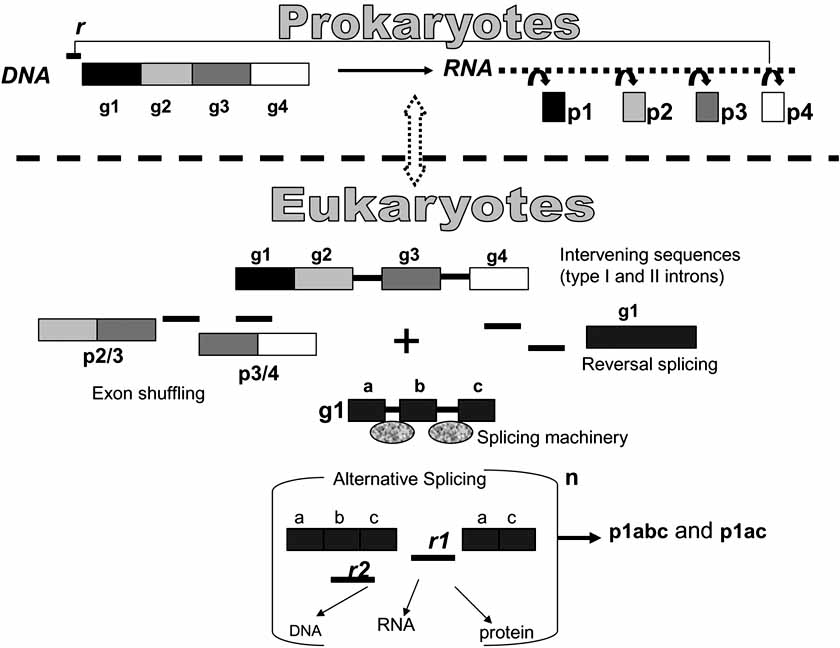
A model illustrating Eukaryotes intron-mediated gene expression versatility. g stays for genes that code for proteins (p). r stays for regulatory elements. The n outside the bracket stays for node which means a DNA locus transcriptionally active. The node is part of a vast gene network, with multiple nodes, that may change anytime during cell life and metabolism. This model should make it appreciable that the presence of introns in Eukaryotes may contribute to the increase of products and regulatory factors without altering the number of the coding genes (four in this example). Eukaryotes versatile expression has been gained in the course of evolution through the occurrence of different events such as the inclusion in protein coding genes of intervening sequences capable of self-splicing (groupI and II introns), exon shuffling, reversal splicing and the entry of the nuclear spliceosome. This latter has contributed to release those sequence constraints present in self-splicing introns. As a consequence, spliceosomal introns increased their sequence variability and may have acquired novel trans-acting regulatory functions. On the opposite, Prokaryotes have maintained their linearity of expression, substantially supported by monocistronic RNAs and few products endowed with simple-circuited regulatory functions.

The reason why, despite massive losses in some branches of the tree, introns have been maintained in the course of evolution, to the extent that a significant proportion of intron positions is conserved across the millions of years separating plants from animals [10], could hardly be explained without a functional role. Similarly, regulatory roles are likely to be played by a large part of noncoding DNA, mainly intergenic sequences and antisense sequences, that have been recently found to be transcriptionally active in both bacterial and eukaryotic cells [11]. This genomic DNA that definitively codes for a large amount of transcripts of unknown function (TUF) [12] has gained the captivating name, of “the dark matter” of the genome. Between 60 to 70% of the human genome has recently been estimated to be transcribed in one or both strands [13]. To this regard, it has been proposed that introns and other ncRNAs, have evolved to constitute a network of controlling molecules that co-ordinately regulate gene expression through multiple interactions with other molecules such as DNA, RNA and proteins. This network would represent the real gain with respect to the linearity of genetic information that is assembled in the genomes of prokaryotes. The network could explain why the increase in organism complexity can occur without an exponential increase in the number of protein coding sequences [14] (Fig. **1**).

Assigning regulatory functions to the ncRNAs, that represent something like 98% of the transcriptional output of a human cell, also helps to explain the G-value paradox, emerged from genomic and transcriptomic projects. It has been shown that the estimated number of protein coding genes does not correlate with the organism complexity as it was previously presumed: i.e. humans and *C. elegans* have roughly the same number of protein coding genes [15]. This inconsistency can be explained by the observation that organism complexity does better correlate to the proportion of noncoding DNA [2**].

Nowadays we are witnessing an epochal shift with regard to the genome fundamental unit: the previous proteo-centric view is being replaced by a more widely distributed and multifunctional model that is centred on transcripts [12].

Introns represent a fraction of the transcriptome to which different regulatory functions have been already assigned and new ones are likely to be added in a short time. In plants, introns have mainly been investigated for their role in gene expression, since they are capable of increasing and addressing it. Hereafter, we will briefly review the more recent information about splicing in plants, to then discuss the different roles played by introns in the light of most recent information. These latter can be summarized as follows:

1. Introns, especially first introns, may contain binding sites for transcription factors or may act as classical transcriptional enhancers.
2. In plants, several introns may influence the expression of their own genes by increasing transcript levels. This is frequently observed for the first or second intron of a given transcription unit.
3. Some introns are also responsible for tissue or development specific gene expression.
4. Some introns, mainly first introns, may act as internal promoters to produce alternative mRNAs. When such intronic promoters lay within leader introns, the alternative transcripts differ only for their first, noncoding exon.
5. Introns may release trans-acting factors such as microRNA (miRNA) and small nucleolar RNA (snoRNA).

## SPLICEOSOMAL INTRONS ORIGINS: INTRONS EARLY OR INTRONS LATE OCCURRENCE?

Even if the debate on introns origin is still open, the contraposition between the “introns early” model, sustaining the existence of introns in the ancestors of prokaryotes and eukaryotes [16, 17] and the “introns late” hypothesis, suggesting that introns colonized eukaryotic cells after their divergence from prokaryotes, [18, 19] have reached an equilibrium with the proposition of combinatorial models such as that of the “synthetic theory” of intron evolution [20] or the “compromise solution” [21].

If the presence of introns in LUCA, the putative Last Universal Common Ancestor of all leaving organisms is still debated [22], new data accumulated in recent years have strengthened the suggestion that the common ancestor of all eukaryotes possessed introns and a spliceosome: some intron position and basic spliceosomal RNA and proteins are in fact common to all extant eukaryotes [23].

Extensive studies on eukaryotes genomes aimed to asses the rate of intron gain or loss that has occurred during evolution are often biased by the different assumptions that each author makes followed up with the choice of different datasets and statistical methods. This may sometimes lead to opposite conclusions. Furthermore, some apparently conserved intron positions may actually occur due to parallel insertions at proto-splice sites [24] as early suggested by Dibb and Newmann in 1989 [25]. Nevertheless, a good agreement has now been reached that massive intron loss has occurred in some branches of most lineages, while significant intron gain occurred in the past tens to hundreds of millions of years has been documented for fewer cases [4].

To this regard, very few analyses have been performed on plant genomes and the results are also discordant. A study from Lin *et al. *[26] reported a majority of intron losses over gains in genes from a duplicated region of the rice genome. Opposite results were obtained by Knowles and McLysaght [27] from the analysis of 2563 paralogous pairs in Arabidopsis. They found a majority of intron gains over losses (56 versus 39). In a subsequent study though, most of the same 56 introns were found in the same position in the genomes of tomato and rice and therefore they are more likely to have been lost rather than gained after gene duplication [28]. By analyzing more than 8000 putatively orthologous genes between rice and Arabidopsis, these Authors concluded that massive intron loss dominated plant evolution. By comparison of the intron pattern of six conserved genes for sugar phosphate metabolism between key species of all Plantae lineages, Teich *et al. *[29] concluded that a 500 million years of stasis characterized land plant evolution, while some unique intron position in *Marchantia polymorpha* (a liverwort) may correlate with the transition to terrestrial habitats. Data from orthologous genes in chlorophycean green algae displayed largely different intron patterns.

## SPLICING IN PLANTS: DIFFERENCES BETWEEN MONOCOTS AND DICOTS?

The basic spliceosomal machinery seems to be roughly conserved in all eukaryotes even if many eukaryotic lineages have acquired their own specificities [30, 31].

Data from the two completely sequenced plant genomes of *Arabidopsis thaliana* and rice (*Oryza sativa*) indicate that in both species, about 80% of coding regions contain introns, with a similar intron density of about 4 introns per gene.

Data from the Arabidopsis genome sequencing led to the filing of ASRG (Arabidopsis Splicing-Related Genes database) in which 74 snRNA genes and 395 genes coding for splicing-related proteins have been inserted, based on sequence comparison and motif searches [32]. Splicing regulators such as Serine/Arginine-rich proteins (SR proteins), SR protein kinases and hnRNP proteins are more redundant in plant than in animal cells and include many stress-induced proteins. SR proteins are highly conserved splicing factors, containing one or two RNA recognition motifs (RRMs) at their N-termini and repeated arginine and serine residues at their C-terminal domains. SR proteins are involved not only in splice site recognition, acting as repressors or activators of splicing but are also involved in nucleo-cytoplasmic shuttling. SR family comprises 19 members in Arabidopsis and 24 in rice. 12 of the Arabidopsis SR proteins have their counterparts in metazoans but 7 are exclusive for plants and some bear additional aminoacid domains [33]. Furthermore, different SR proteins derive from alternative splicing, triggered by stress and temperature in a tissue-specific manner.

One of the main differences between splicing in plants and in vertebrates concerns splice site recognition: while vertebrates bear introns that may span hundreds of thousands of base pairs, plants have no more than 1-1,5 Kbp long introns, rarely 2 or 3 Kbp. The exon definition model proposed for splicing in mammals is sustained by the finding of splicing enhancer and silencer sequences within exons, and applies well to those organisms. By converse, plants bear shorter introns whose base composition is strongly different from that of exons (10-15% more AT rich) as it is also observed in invertebrates such as insects or nematodes [34, 35]. AU-rich sequences are essential to define introns in plants [36] and RNA binding proteins with affinity for U-rich sequences have been identified in *A. thaliana* [37]. Nucleotide composition seems to be one of the most striking reasons at the base of the early observation that mammalian introns are not or poorly spliced in a dicot plant system such as tobacco protoplasts [38, 39]. Conversely, maize protoplasts were found to be able to splice some mammalian introns [40]. This is likely due to the different splicing requirements present in monocots with respect to dicot plants. Many monocot introns, such as the maize *Adh1* intron1 (57% AU-rich) are not efficiently spliced in dicot cells [40, 41]. Monocot introns are on average 63% AU-rich, but about 20% of them have higher GC content (50%), while dicot introns are 67% AU-rich and less tolerant to variations in nucleotide composition. In example, addition of T-stretches can lead to efficient splicing of an artificial and otherwise poorly spliced GC-rich intron, in transfected tobacco protoplasts, [42]. Similarly, a synthetic intron, 75% AU-rich with canonical 5’and 3’ splice sites and a branchpoint consensus sequences, is efficiently spliced in tobacco protoplasts, but cannot tolerate a GC-rich sequence insertion that reduces the AU content below 59% [36]. All these observations favor an intron definition model for splice site recognition that is also supported by the high frequency, more than 50%, of intron retention in alternatively spliced transcripts in plants [32]. Even though specific exon sequences required for exon recognition have not been identified yet, a combinatorial model was proposed [43], in which flanking exons also contribute to splice site selection. Increasing the GC content of exons flanking a GC-rich intron of the maize *Bronze2* gene, enhances splicing efficiency while a reduction in the GC content diminished splicing [43, 44]. Further indications for a concomitant recognition of exons during splicing, came from the evidence of exon skipping in Arabidopsis splice site mutants [45].

All these studies have been made on the assumption that rice or maize are good model plants for all monocot species, but recent evidences pose the question on how representative the Poales (Graminae) are for the monocots as a whole. Recent data from onion EST [46] highlighted that Asparagales are more similar to eudicots with respect to their genomic characteristics (i.e. GC content, codon usage, GC distribution) than to Poales. Differences in splicing efficiency have also been highlighted among dicot species: the criptic intron of the GFP gene of *Aequorea victoria* was identified due to the poor expression of the reporter gene in transgenic Arabidopsis plants. The same sequence in tobacco is spliced with 40% efficiency, implying differences in splice site recognition between these two plant species. These observations demonstrate that the plant kingdom is much more differentiated at the molecular level than previously thought and inference from one or two model species to larger phylogenetic groups is not straightforward. In other words, working on plant species from both distantly related and lower phylogenetic groups is also important to gain a more detailed view of plant complexity.

## ALTERNATIVE SPLICING: INTRONS AS EXONS

Alternative splicing (AS) accounts for the discrepancy between the number of putative coding genes in sequenced genomes and the larger number of EST found in eukaryotes. Together with alternative transcription start sites, this mechanism is responsible for generating even tens of different transcripts from one gene, leading to the production of different or slightly different proteins.

AS is common in higher eukaryotes and increases with organism complexity: it is rare in unicellular organism and in fungi. In extreme cases, one gene can lead to the production of hundreds of alternative transcripts as reported for the cell-specific expression of 576 alternatively spliced forms of K+ channel mRNA in sensory-receptor cells of the inner ear of birds or for the 38,000 different mRNA isoforms transcribed from the Drosophila* Dscam* gene that codes for the axon guidance receptor (reviewed by [47, 48]).

In plants, the phenomenon was considered scarcely relevant, but is now emerging as important as it is in animals [49]. Several examples of AS events leading to different proteins, such as Rubisco activase, Arabidopsis *FCA* gene, have been recently documented in plants [50], but this is likely to be the tip of an iceberg.

The most recent estimation of the number of alternatively by spliced genes is about 20% for both Arabidopsis and rice but this number may even increase since estimation in humans has recently raised to more than 60% [32]. From these computational analyses, Intron Retention (IR) resulted to be the most common AS event in Arabidopsis and rice, accounting for about 40-45% of total [32, 51] but the least common event in humans, where Exon Skipping prevails. This finding is an agreement with the predominant mode of splice site recognition typical for the two cases: failure to recognize splicing signals would lead to exon skipping in mammals, where an exon recognition mechanism is predominant, and to intron retention in plants, where an intron recognition mode is adopted.

In the case of intron retention, the functional significance of AS is not so evident and leaves the doubt that incorrectly spliced transcripts constitute a background of “escaped” mRNA that are not eliminated by the cell. However this is unlikely, since most retained introns within coding sequences contain stop codons, supposed to drive mRNAs to Nonsense mediated decay (NMD). Furthermore, in some cases it has been demonstrated that intron-containing transcripts are not retained in the nucleus (as they should be in case of incomplete or inefficient splicing) but are found associated to polyribosomes [52].

Alternatively, introns may be retained to play a functional regulatory role. In vertebrates, it has been suggested that the coupling of AS and NMD could be an important post-translational mechanism to adjust the level of transcript isoforms [53]. Indirect indications suggesting a role for retained introns came from the fact that about 30% of intron-retaining transcripts in Arabidopsis are also conserved in rice [32]. A regulatory role for intron-containing mRNAs fits well with recent data from cDNA cloning and genome tiling experiments, indicating widespread transcription of the majority of genome on one or both strands in mammal [11] and also in plant [54] DNA. This results in the regulated production of huge amounts of transcripts of unknown functions (TUFs), that are devoid of any protein coding potential and derived from intragenic regions, pseudogenes or from gene noncoding strands. TUFs represent the “dark matter” of the genome and constitute a hidden and additional layer of control for gene expression [12, 55, 56].

Introns in 5’-and 3’UTRs are more commonly retained than those constitutively spliced. This is likely to have a regulatory meaning since UTRs are important regulators of gene expression by influencing mRNA stability and translational efficiency. This can occur through the presence of upstream ORFs, the presence of secondary structures or binding sites for tissue-specific proteins or short RNAs like miRNA and short interfering RNAs [57, 58]. Multiple UTRs enable tissue-specific protein expression without influencing overall transcription and alternative UTRs may trigger tissue-specific responses to physiological stimuli [58].

In plants, AS is regulated in a tissue-specific manner and is strongly influenced by stresses and hormones. The regulation of alternative splicing is quite complex and involves splicing regulators, such as SR proteins, protein kinases, hnRNPs, probably driving it in a concentration-dependent mode. The synthesis of splicing regulators is in turn controlled by stress-dependent alternative splicing so that environmental stimuli may control the AS pattern of co-ordinated sets of related genes [59**]. In animal systems, there are indications suggesting that trans-acting RNA molecules are involved in splice site selection [48, 56, 60].

Furthermore, not only protein coding RNA, but even noncoding RNAs, may be alternatively spliced, as it is the case for the recently discovered NRON RNA [61] a repressor of the transcription factor NFAT that shows a distinct tissue-specific distribution of its spliced variants. RNA interference experiments have shown that this ncRNA is likely involved in the nucleo-cytoplasmic transport of NFAT and possibly of many other proteins.

## ALTERNATIVE FIRST EXONS AND INTRONS AS PROMOTERS 

In a proteo-centric world, AS is a mechanism that increases the number of protein isoforms encoded by genes supporting the synthesis of different or slightly different products. Although undoubtedly true, AS is not limited to this output. In fact it is frequently found that splice variants also produce different transcripts with the same coding sequences. This is the case for retained introns within 3’ or 5’ UTR or for transcripts with alternative first noncoding exons. Furthermore, 30-40% of AS in Arabidopsis leads to premature stop codon formation, targeting transcripts for NMD.

In the human genome, it has been estimated that 10-18% of genes express alternative 5’UTR by the use of multiple promoters [62]. Recent computational analyses indicate that the phenomenon is likely to be underestimated [63, 64].

An analysis of 91,425 rice 5’ end ESTs detected 46 (4%) potential Alternative First Exon (AFE) clusters. RT-PCR analysis of 12 of these clusters, in six different tissues and stages, revealed tissue-specific expression of the alternative first exon for 5 of them.

**Fig. (2):**
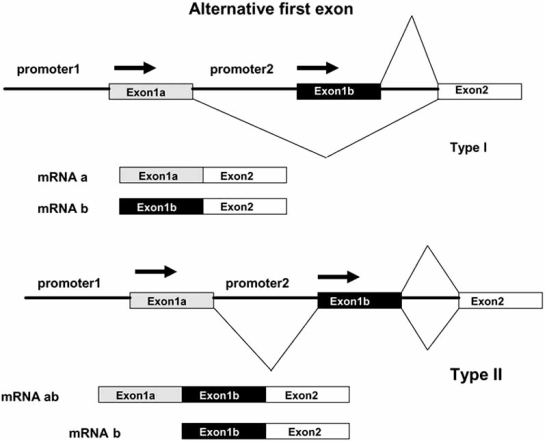
Schematic representation of the two mechanisms by which AFE- containing transcripts are generated (see text).

A more recent work identified about 5-6% of AFE clusters in both rice and Arabidopsis [64]. Many of them produce proteins with alternative N-terminal regions but a significant proportion, 50% in rice and 19% in Arabidopsis, lead to transcriptional variants differing only for their 5’UTR. AFE may be produced by two mechanisms (Fig. **2**): in the first (type I), the two first exons are mutually exclusive; in the second (type II), the first exon of an alternative mRNA, exon 1b in Fig. (**2**), falls internal to the other.

When alternative exons are distant (more then 500 base pairs) they are usually associated with alternative promoters, the most downstream of which lays within an intron. Estimations from available datasets indicated that about 58% of rice and 23% of Arabidopsis AFE containing genes may be derived from alternative promoters. 

The functional significance of splice variants encoding the same protein is likely to be merely regulative and may occur for the following reasons:

1. alternative promoters may vary for strength, tissue or condition-specificity thus binding different transcriptional factors or repressors.
2. mRNAs with different 5’UTRs may differ in stability or translational efficiency and this may cause a finer tuning of expression levels, i.e. they may contain uORFs that reduce correct translation. 5’UTR may also be the target for regulatory miRNAs (see below).
3. the structure of loci containing alternative promoters is such that the downstream promoter may lay within an intron sequence that is spliced from the upstream transcript. Hence, alternative first exons mean alternative introns. Since plant introns can specifically influence expression at post-transcriptional level, this latter may obviously be a mechanism by which gene regulation is further influenced. Promoter function is thus an additional function for introns located in the 5’ region of a gene. To this regard, the following few examples have so far been reported in plants.

Genes encoding pyruvate orthophosphate dikinase (PPDK) from maize [65] and Arabidopsis [66] produces two alternative transcripts from two independent promoters. This results in two N-terminal variants of the protein that are differentially located, one in the cytosol, and the other targeted to the chloroplast. SYN2 of Arabidopsis [67] is a protein required for chromatin condensation. Two transcripts are synthesized starting from two alternative promoters. They differ for the first exon, each containing its own ATG and lead to the production of two proteins that differ in their N-terminal domain. The transcript synthesized from the upstream promoter is expressed at a low level in many tissues, while the alternative transcript shows high level of expression restricted to buds. The specificity of expression may be due to either the alternative promoter or to the information content of the intron.

Bean *PvSR2* [68] is a heavy metal inducible gene. It contains a weaker promoter within the first intron present in the ORF. This alternative promoter is not inducible by heavy metals and leads to the production of a different protein.

Furthermore, similar data were reported for two unrelated rice genes, *Ostub4* and *CDPK2*, the first encoding for a beta tubulin isotype, the second for a Ca++-dependent protein kinase [69-70]. While in all the examples mentioned above, alternative promoters lead to the production of slightly different protein variants, in both the *Ostub4 *e *CDPK2* genes the alternative first exons were noncoding ones, so that the protein products from the alternative transcripts remained the same. In the case of *Ostub4*, the intronic promoter showed a very low activity in transient transformation assays, while the upstream promoter sustained high level of GUS activity [69]. This latter was also capable to sustain high levels of GUS expression in transgenic plants, only when associated to the leader intron (Gianì *et al*. 2008, submitted)^1^. In the case of *CDPK2*, the intronic promoter has a strong activity, but only in the presence of an intron located further downstream [70]. Under this configuration it is able to sustain tissue-specific activity in transgenic plants. The upstream promoter has not been isolated and tested yet.

In all these cited examples, intron sequences may probably exert their function at different levels, since they can act as transcriptional promoters and as intronic enhancers, probably by a post-transcriptional mechanism (see next paragraph).

## INTRONS AS GENE EXPRESSION ENHANCERS 

Recently, after years of “promoter leadership” that followed the discovery of transcription factors, the involvement of other regulatory sequences in gene expression has rapidly emerged, revealing that all the downstream elements present within transcribed sequences (5’ and 3’ UTRs, exons and introns) may also play an important role in gene expression contributing to a variety of post-transcriptional regulatory mechanisms [71].

In the case of introns, the general observation that intron-containing DNA sequences are better expressed than their cDNA counterparts was originally made in mammalian cells [72] and later confirmed in invertebrates [73] and plants. The effect of introns on mRNA expression can be explained by the tight and complex interplay between the spliceosome and the hundreds of proteins of the “mRNA factory” that are involved in all the steps of transcript synthesis and maturation such as the formation of the transcription complex, capping proteins, and nuclear pore components [74]. So, splicing as a whole can enhance gene expression through feedback actions on mRNA transcription and maturation [75], or by imprinting the mature transcript for enhanced translation, although such mechanisms have not been elucidated yet.

In plants, early observations on the effect that some introns of maize genes (*Adh1, Bz1, Act1, Shrunken1*) have on gene expression, date back to the late eighties. The inclusion of such intron sequences in reporter-containing expression plasmids greatly enhanced downstream reporter gene expression [76-78]. However, the effect was somehow intron-specific since introns lacking enhancing effect were also described, i.e. the pea phaseolin intron [79], the maize *Adh1* intron 9 and *Hsp81* intron 1 [80, 81] and the first introns of the Arabidopsis profilin 4 and 5 genes [82]. The inclusion of enhancing introns in association with their respective promoters, such as the maize ubiquitin1, is widely employed in plant expression vectors to increase foreign gene expression in transgenic systems [83]. Since its discovery, several investigations have been performed to understand the molecular basis of the Intron Mediated Enhancement of gene expression (IME), as this phenomenon was then called [80]. Experiments have mainly been performed by transient transformation assays or by production of transgenic plants. Enhancing introns have been found in many genes from both monocot and dicot species, and also from non vascular plants such as the moss *Physcomitrella patens* [84]. It has turned out that the observed degree of enhancement may differ widely, from two-threefold to one hundred fold and that many factors can influence IME such as: nucleotide composition of the intron and of the flanking exons [77], the reporter gene used [85], the promoter employed [78] and the position of the intron [86], thus highlighting the complexity of the phenomenon.

Discordance between some of the reported results may be partly due to the different experimental systems adopted and also to the fact that plasmid manipulation may bring to undesired modifications that can ultimately influence gene expression. Nonetheless, experimental data support the conclusion that the cause of IME is not unique. On the contrary, introns may act in a number of different ways, also depending on the different gene context. Accordingly, regulatory elements within a transcribed sequence (5’ and 3’, exons and introns) may selectively act at either DNA or RNA level. As recently discussed by Mattick and Makunin [56], RNA molecules bear features of both analogic (based on structure) and a digital (based on its sequence) signal that may act in *cis* and in *trans*, as a sensor or a transmitter.

Some plant introns are likely to act at the DNA level since they can function independently of their position and orientation, i.e. Arabidopsis *ACT1* [87], and some other introns as in the *AGAMOUS* gene of Arabidopsis (*AG*, see below) have been shown to contain enhancer-like transcriptional elements, but in most cases, the presence of introns within the transcription unit (like maize *Adh1* introns 1, 2 and 6 [76, 80], rice *OsTua1 *[88]), and in the sense orientation (like maize *Adh1 *and* Sh1* first introns [78] and petunia *PhADF1 *[89]), is chiefly required for IME. In almost all of the cases analyzed, the presence of the intron increased the steady-state level of mRNA. Nuclear run-on assays, performed on mRNA extracted from Arabidopsis plants transformed with plasmids bearing the PAT1 first intron upstream of GUS showed no increase in the transcription rate [86]. These results favor a post-transcriptional mode of action, even if a feed-back effect on transcription rate cannot be ruled out.

Further effects on translation have been suggested in one case where no increase in the mRNA level was observed [90] and in others where the mRNA enhancement did not correlate to the increase in the protein level or activity [82;91]. A very important issue raised by these experiments concerns the possible dependency of IME on splicing. Mutagenesis of splice recognition sites that completely impair splicing, abolished IME for some introns such as those of the maize *Adh1*, *Hsp82* and *Sh1* genes [77, 81, 92] but not for intron1 of the At PAT1 gene [93].

The position of the intron also appears to be an important factor. Insertion of intron 1 of the* Pat1* at different locations within the transcribed sequence greatly influences IME: longer is the distance from the 5’ –end of the gene, lesser is the effect [94]. Similar results were found for the *Sh1* leader intron [80] and for the rice triosephosphate isomerase *Tpi* first intron [95]. Three different introns from the maize *RpoT* gene, while all efficiently spliced, have different enhancing effect when placed in different positions within the *luciferase* coding sequence with some of these locations that could even cause negative effects [90].

In any case, splicing per se does not seem to be sufficient for IME to occur since the degree of expression enhancement varies greatly in relation to the intron used. In fact, as already mentioned, it has also been reported that some correctly spliced introns fail to exert any effect. *PhADF1* intron1 is required for detectable gene expression in transgenic tobacco. If replaced by intron 2 of the same gene, the level of expression falls below that observed with a construct that lacked introns [89].

In most cases, large internal intron deletions are tolerated without significant effects on IME [77, 88], suggesting the lack of a strict sequence requirement or redundancy of important regions. Internal deletions of the maize *Shrunken1* leader intron extended up to 85% of the sequence, but preserving the splicing sites, can still support the same enhancement of expression as observed with the intron in the native configuration, but a stretch of Ts, not required for splicing, is necessary for enhancement [93]. Conversely, progressive deletions of the intron of the *AtEF1-A3* seem to reveal a correlation between intron length and intron-mediated enhancement [96]. Deletion of internal parts of the *PhADF1* first intron suggested that the 5’ distal region is more important for enhancement than the intron 3’ region [82].

So, despite the many informations that have been obtained up to now, more experiments with additional and specific promoter-intron combinations are required to reach a better understanding about the mechanisms underlying intron-mediated enhancement of gene expression, in an effort to identifying the true determinants.

Ultimately, it should be noted that the requirement of introns for gene expression may be different in monocots compared to dicots and differences in these latters can also be scored. The first intron of the castor bean catalase gene (*cat-1*) driven by the 35S CaMV promoter, can enhance GUS expression in rice protoplasts and leaves, while it has no effect in transgenic tobacco plants. This finds explanation with the fact that the intron was efficiently spliced in rice but not in tobacco [97]. This is in accordance with the different splicing efficiency of a GFP cryptic intron reported in tobacco and Arabidopsis.

Similarly, the upstream sequence of the rice *Tpi* gene can direct GUS expression in transgenic tobacco plants in the absence of the first intron, that is instead required for expression in rice. Conversely, the presence of the intron impairs expression in tobacco [98]. 

## INTRON DEPENDENT SPATIAL EXPRESSION (IDSE)

The effect exerted by some introns on downstream gene regulation may not be restricted to the level of expression, but can also influence the actual site of expression. Such evidence comes from expression studies carried out in transgenic plants transformed with different combinations of plant promoters and reporter genes. It was reported that the *in vivo* pattern of tissue-specific expression can also depend on introns.

Early evidence for the need of intragenic sequences for tissue-specific expression in plants came from works on sucrose synthase *Sus3 *and *Sus 4* genes in potato [99,100] and from expression studies on the *AGAMOUS *(*AG*) gene of Arabidopsis [101].

Studies performed on the *AG* promoter indicated that the pattern of GUS expression driven by a 6 kb upstream sequence in transgenic Arabidopsis plants did not match that obtained from *in situ* hybridization experiments. The addition of a 3.8 kb intragenic sequence inclusive of the first two introns restored a correct expression pattern with overlapping profiles between the two experimental approaches.

A subsequent work clarified the original finding since it demonstrated that enhancer-like elements that specifically regulate expression in carpels and stamens were found in the large second intron of the AG gene. A correct pattern of expression was also maintained by inserting this intron in the unstranscribed region of the gene, independently of its orientation [102].

*Sus3 *and* Sus4*, two sucrose synthase genes from potato, bear long introns within their leader sequences. It has been demonstrated, both in transgenic potato and tobacco plants, that such introns are necessary for the correct level and site of expression of a downstream reporter gene. Sus4 leader intron is required for high-level tuber expression and for sucrose induction. Introns contain both positive and negative regulatory elements, whose deletion caused either ectopic expression or absence of expression. Whether their regulatory role is exerted at the transcriptional or post-transcriptional level was not investigated.

In another case, that of rice *Ostuba1*, the effect was exerted at level different than DNA. The first intron of the coding region of rice *OstubA1* was found to be necessary for the correct expression pattern of GUS in transgenic rice plants. In its absence, GUS expression shifted in roots from the apex to the elongation zones, while in leaves became diffusely distributed rather than being confined to the innermost of leaf sheaths. In this case the enhancing effect was lost when the intron was inserted in the 5’ untranscribed region or after the GUS sequence. Similar data have been confirmed for the cognate *Ostua2* gene and for rice beta tubulin gene *Ostub4* (Gianì *et al., *2008, submitted)^1^.

Arabidopsis *ACT1* gene is mainly expressed in pollen and ovules, although expression in meristematic regions of roots and shoots is also observed. In transgenic Arabidopsis plants, the precise deletion of the whole leader intron from a chimeric ACT1::GUS gene, abolished expression in all tissues while reducing pollen-specific GUS activity to about 10% [88]. The same study reports the interesting possibility that the same intron may have dual functionality. Positioning of the leader intron, in both orientations, upstream of the promoter region, fully restored high level expression in pollen, but not in any other tissue, suggesting an enhancer-like effect for pollen expression and a more classical IME effect for expression in meristematic tissues and ovules. Conversely, the replacement of *ACT1* leader intron with that of *ACT2*, an actin gene mainly expressed in vegetative tissues and not in reproductive organs, can rescue activity in meristematic tissues but not in pollen. This indicates that pollen-specific elements are contained within the ACT1 leader intron sequence.

A recently investigated case is that of the Arabidopsis profilin gene family members: replacing the first intron of the reproductive *PRF5* gene with that of the vegetative *PRF2* isotype, completely re-directed expression driven by the *PRF5* promoter in transgenic Arabidopsis from an anther-specific to a constitutive, generic pattern [103]. Similar findings were obtained by the same Authors by replacing the first intron of the *PRF5* gene with that of the petunia actin-depolymerizing factor1 gene (*PhADF1*): the intron re-addressed GUS expression to vegetative tissues (Jeong *et al. *2007).

## INTRONS AS A SOURCE OF REGULATORY RNA

Last but not the least, spliceosomal introns can influence expression by encoding for trans-acting regulatory RNAs, such as intronic miRNA, snoRNA and others RNA molecules that are processed from intron precursors by specific RNAse/protein complexes. The issue of short regulatory RNAs such as short interference RNAs (siRNA) and micro RNAs (miRNA) represents a very recent yet greatly expanding field of investigation. Many review works have thoroughly analyzed the state of the art including plants [104-106**]. Here, what we like to stress is the intron-derived origin of many short RNAs and the possibility for introns of encoding many more of these regulatory transcripts.

Small nucleolar RNAs (snoRNAs) are the oldest family of short RNAs. They are short RNAs assembled in ribonucleoprotein complexes whose function is the nucleotide modification of ribosomal and spliceosomal RNAs [107]. Almost all known snoRNAs in animals are derived from introns of coding or non-coding mRNAs. Recent evidence suggests that they can target other RNAs and that they play additional functions in alternative splicing, as it was shown for the aberrant splicing of the serotonin receptor 2C gene, the likely cause of the Prader-Willy syndrome [60].

miRNAs are short single-stranded 20-24 nucleotide long RNAs first identified in *C. elegans. *They work by base-pairing to mRNAs, targeting them to degradation or inhibiting translation. They derive from longer precursors, cleaved by two type III RNAse/protein complexes, Drosha and Dicer. Again, a great number of miRNAs is processed from intron precursors, and their synthesis is regulated contextually to the gene in which they are hosted [108]. This subclass of miRNAs relies on RNA polymerase II and the spliceosome for their biogenesis, produced as part of their hosting transcription units. Very recently, intronic miRNAs have been demonstrated to induce RNA interference in mammalian cells [109*].

A growing number of miRNA species have been also found in plant cells, many of which are conserved between distantly related species [110], while others are lineage-specific. Dicer and Drosha have their plant counterparts in *CARPEL FACTORY* and *DICER-LIKE* (DCL) genes. Mutations in *CARPEL FACTORY* indicate that miRNAs may play pivotal roles in plant development [104-106]. miRNA targets include transcription factors and genes involved in stress response, hormone signalling, and cell metabolism. 

Of recent, miRNAs that directly originate from debranched introns (mirtrons), bypassing Drosha processing whose function is replaced by the spliceosome, have been identified in *Drosophila* and *Caenorhabditis* [111*]. Mirtrons have not been identified in plants or in mammals yet. However, a number of plant introns present the length prerequisite (about 60 nucleotides) for an intron to become a mirtron.

The existence in the human transcriptome of long, unspliced, antisense intronic transcripts can reveal a new class of regulatory RNAs. Antisense intronic transcripts whose level is regulated by androgens in a prostate cancer cell line have been recently reported [112]. The general feeling is that introns may hide new unknown functions since there is good evidence that intronic RNAs may actually be processed to smaller RNAs with significant half-lives and specific subcellular locations [113].

## INTRONS AS A SOURCE OF POLYMORPHISM

At the beginning of this review we have reported on how introns are being used as key elements for molecular evolution studies, the aim of which is to identify LUCA, the Last Universal Common Ancestor of the living organisms. These studies are mainly based on the analysis of eukaryotic genomes with respect to intron conserved position rather than sequence composition. This latter is in fact quite variable and makes it difficult to identify conserved nucleotide motifs in paralogous or orthologous intron pairs. Whatever is the identity of LUCA, it is a fact that introns are largely abundant in Eukaryotes where they contribute to increase the variability of protein isoforms, mainly through the mechanism of AS. We have also learnt that AS can lead to the production of different transcripts coding for the same product and that introns may be part of non-coding exons. Moreover, introns can host promoters and transcriptional start sites. Introns influence gene expression through IME and IDSE and are the elements from which miRNAs and snoRNAs are produced. Due to all this bundle of regulatory functions, it should not come to surprise that introns length and sequence composition can be highly variable and that no appreciable significant nucleotide homology can be found when comparing introns of orthologous genes and gene-families. At the same time, the position of introns is not randomly scattered within genes and across the genomes. On the contrary, intron position is amazingly retained across unrelated eukaryotic species [22, 23]. This combination of intron positional retention and intron sequence and length variability provides the basis for an easy, fast and yet informative method of detection of DNA polymorphisms that is called ILP for Intron Length Polymorphism [114]. This method is particularly successful and informative for those introns that are part of genes or gene-families encoding for housekeeping functions such as products involved in cell division or cell architecture. In this case, the difference between nucleotide sequence conservation of the exons and sequence variability of the introns reached its apex and offers the best of exploitation. This is true to such an extent that it could be proven that plant species can be simply defined by theirs specific ILPs. In other words, the emergence of a new species is accompanied by its specific panel of ILPs. To a first approximation, ILP markers can be envisioned as true DNA barcode. Our laboratory can certainly provide evidences for this strong statement based on the use of the two introns present in the coding sequence of the vast majority of the plant beta-tubulin genes [115, 116]. These two introns are in fact used as a combined source for species and subspecies characterization. Each species we have analyzed, being dicot or monocot, gymnosperm or angiosperm, shows its specific ILP pattern that is associated to variability in the members of its own beta-tubulin gene family. We have named cTBP, for combinatorial Tubulin Based Polymorphism, this specific ILP.

ILP markers have several advantages, the most important of which is transferability across the plant species. It has been shown that out of 51 pairs of rice-based ILP primers, only 6 of them (11.8%) failed to generate PCR products from genomic DNA extracted from 8 different plant species while 24 pairs (47.1%) yielded similar amplification products as observed in rice [114]. As just mentioned, our cTBP primers have successfully worked in the amplification of species-specific tubulin introns in no less than 20 different plant species insofar. This means that species/subspecies identification and characterization can be easily achieved with the use of a common set of primers, in a day of work, with reproducible results. In addition, information on the molecular evolution of introns is readily available. Besides interspecies transferability, additional advantages of the ILP markers are their codominancy, neutrality and stability. All these features can be of use in rapid gene isolation rather than in the assistance of crop breeding.

## CONCLUSIONS

It is clear that introns have regained general interest by virtue of their recently uncovered multi-functionality. This also explains why they have been conserved along great evolutionary distances.

While positioning within genomes has been largely maintained, nucleotide sequence has substantially diverged with the exception of short conserved elements responsible for intron specific functions (i.e. miRNAs formation). Introns can regulate the level and the pattern of gene expression during plant growth and morphogenesis and under different environmental conditions. 

Introns are key elements of the new molecular genetics, dealing with unannotated TUFs and the role they have in the general control of eukaryotic gene expression.

## Footnotes

1 Gianì S, Altana A, Campanoni P, Morello L, Breviario D. IME and IDSE (Intron Dependent Spatial Expression) regulate the expression of rice α-  and β-tubulin genes (submitted, 2008).

## ABBREVIATIONS

IME: = Intron Mediated Expression
IDSE: = Intron-Dependent Spatial Expression
NMD: = Nonsense Mediated Decay
AS: = Alternative Splicing
AFE: = Alternative First Exon
ES: = Exon Skipping
IR: = Intron Retention
ILP: = Intron Length Polymorphism
UTR: = UnTranslated Region
ORF: = Open Reading Frame
TUF: = Transcript of Unknown Functions
LUCA: = Last Universal Common Ancestor
